# False positive technetium-99m pyrophosphate scintigraphy in a patient with cardiac amyloidosis light chain: Case report

**DOI:** 10.1097/MD.0000000000025582

**Published:** 2021-04-30

**Authors:** Yu Zeng, Timothy J. Poterucha, Andrew J. Einstein, Qing Zhang, Yucheng Chen, Hangyu Xie, Ke Wan, Yujia Liang, Juncheng Chen, Gongshun Tang

**Affiliations:** a Nuclear Medicine; b Seymour, Paul, and Gloria Milstein Division of Cardiology, Department of Medicine; c Department of Radiology, Columbia University Irving Medical Center, New York, New York; d Cardiology, Cardiac MR; e Echocardiology, Sichuan University West China Hospital, Chengdu, Sichuan, China.

**Keywords:** cardiac amyloidosis, case report, nuclear medicine, pyrophosphate

## Abstract

**Introduction:**

Patients with cardiac amyloidosis light chain (AL) present with negative Tc-99m pyrophosphate (PYP) scintigraphy (absent or mild heart uptake). On the contrary, patients with cardiac amyloidosis transthyretin (ATTR) present with positive Tc-99m PYP scanning (intensive heart uptake). We present a false positive Tc-99m PYP scintigraphy (grade 2, the heart-to-contralateral ratio is 1.65) in a patient with AL.

**Patient concerns:**

A 42-year-old Chinese man complained of effort intolerance, chest discomfort, and short of breath progressively over 1 year. New York Heart Association Class III. Physical examination showed legs swelling. Laboratory revealed elevated brain natriuretic peptide of 23,031 ng/mL (0–88) and Troponin-T of 273.4 ng/mL (0–14).

**Diagnosis:**

Cardiac amyloidosis light chain. Evidences: free light chains (FLCs): decreased serum free kappa/lambda ratio of 0.043 (0.31–1.56). Immunofixation electrophoresis: a positive lambda light chain monoclonal protein. Cardiac biopsy: HE: Ambiguity Congo red strain. Myocardial immunofluorescence: positive lambda light chain. Myocardial immunohistochemistry: positive lambda light chain, negative kappa light chain, and TTR.

**Interventions:**

Furosemide 40 mg qd, torasemide 20 mg qd, spirolactone 20 mg qd, potassium chloride 10 mL per 500 mL urine, atorvastatin calcium tablet 20 mg qd, aspirin enteric-coated tablets 100 mg qd during the 2-weeks in-hospital.

**Outcomes:**

The patient died 2 months later after discharge.

**Conclusion:**

False positive Tc-99m PYP scintigraphy may rarely presented in patients with cardiac amyloidosis light chain. So, the clonal plasma cell process based on the FLCs and immunofixation is a base to rule out AL cardiac amyloidosis when we interpret a positive Tc-99m PYP scintigraphy.

## 1 Introduction

Systemic amyloidosis is a clinical syndrome characterized by the extracellular deposition of misfolded proteins in various organs.^[1]^ Cardiac amyloidosis occurs when amyloid fibrils deposit within the myocardial extracellular space, causing structural and functional changes, which is characterized by heart failure. The commonest 2 precursor proteins that deposit in the heart are monoclonal immunoglobulin light chain and transthyretin.^[2]^

Patients with cardiac amyloidosis light chain (AL) present with abnormal free light chains (FLCs) and a negative technetium-99m pyrophosphate (Tc-99m PYP) scintigraphy, with an absent or mild Tc-99m PYP uptake in the heart. Patients with cardiac amyloidosis transthyretin (ATTR), including wild-type ATTR (ATTR-wt) and mutant-type ATTR (ATTR-m), present with normal FLCs and a positive Tc-99m PYP scintigraphy with an intensive Tc-99m PYP uptake in the heart.^[3,4]^ However, patients with AL rarely present with false positive technetium-99m 3,3-diphosphono-1,2-propano-dicarboxylic acid (Tc-99m DPD) or technetium-99m hydroxymethylenediphosphonate (Tc-99m HMDP) scintigraphy.^[5–7]^ We present a case of AL who presented a false positive Tc-99m PYP scintigraphy.

## 2 Case presentation

The ethics committee of West China Hospital approved the study. A 42-year-old Chinese man complained of fatigue, effort intolerance over the past year. Six months ago, he presented with chest pain, tachycardia, short of breath, and was diagnosed to be heart failure with estimated New York Heart Association Class III. Echocardiology outside hospital suggested hypertrophic cardiomyopathy. Four months ago, he presented with hepatomegaly and chronic gastritis secondary to heart failure. Furosemide and spironolactone were administered for his heart failure, and clopidogrel sulphate and mosapride were administered for symptomatic treatment. Two months ago, the patient presented right limbs disturbance, and the brain MR imaging demonstrated stroke in his left temporal and insula. He also presented with paroxysmal nocturnal dyspnea, legs swelling, CT-evidenced ascites, and cholecystitis. He was then treated for his heart failure with furosemide and spironolactone, and for his stroke with edaravone, nimodipine. He had no history of hypertension, diabetes, hyperlipidemia, and smoking. His family history was unremarkable. Review of systems did not reveal history of cancer, asthma, prior surgery.

Physical examination on admission: blood pressure, 124/97 mmHg, heart rate, 85 beats/min. Temperature, 36.5 °C, respiratory rate, 22 beats/min, oxygen saturation, 95% without oxygen supplementation, weight/height, 60 kg/165 cm. Itching flat erythema was seen on his hip without subcutaneous hemorrhage. A 3+ cm jugular vein distention was seen. His cardiac border was larger than normal on palpitation, and he had a normal heart rate and rhythm with dual heart sound without murmurs and gallops on auscultation. He had rales at the bases of his lungs bilaterally. He presented with an abdominal distension with shifting dullness, a smooth liver edges on palpation without tenderness or rebound tenderness, and no obvious mass. On peripheral examination, he had a palpable edema on bilateral lower extremities. His neurological examination was unremarkable.

Laboratory on admission: The cardiac biomarkers revealed an elevated brain natriuretic peptide (BNP) of 23,031 ng/mL (0–88) and Troponin-T of 273.4 ng/mL (0–14). FLCs testing revealed a normal serum free kappa light chain of 11.1 mg/L (6.70–22.40), an elevated serum free lambda light china of 257 mg/L (8.30–27.00), and a decreased free kappa/lambda ratio of 0.043 (0.31–1.56). Immunofixation electrophoresis demonstrated a positive lambda light chain monoclonal protein, and negative IGG-KAP, IGG-LAM, IGA-KAP, IGA-LAM, IGM-KAP, IGM-LAM. Electrophoresis showed a normal M-protein of 6.60% (10.6–23.5%).

The results of his blood cell counts were normal; his urinalysis, including cell counts, urine protein quantitative, were unremarkable. The serum electrolyte test revealed a lower serum sodium of 132 mmol/L (135–148) and normal serum potassium, chlorine, magnesium, calcium, and phosphorus. His liver function, renal function, glucose, blood lipid level, coagulation function tests were unremarkable. Table 1.

**Table 1:** Laboratory.

Laboratory parameters	Value	Reference
Albumin	38.4 g/L	40–55
Globulin	21 g/L	20–40
A/G ratio of	1.83	1.2–2.4
Aspartate aminotransferase	39 IU/L	<40
Creatine kinase	103 IU/L	19–226
Alanine transaminase	84 IU/L	<60
Lactic dehydrogenase	350 IU/L	120–250
Serum creatinine	101 mmol/L	53–140
Glucose	5.13 mmol/L	3.9–5.9
Triglycerides	1.36 mmol/L	0.29–1.83
Cholesterol	2.57 mmol/L	2.8–5.7
High-density lipoprotein	0.67 mmol/L	>0.9
Low density lipoprotein	1.3 mmol/L	<4
Estimated GFR	78.67 mL/min/1.73m^2^	56–122
Creatine kinase isoenzyme	4.26 ng/mL	<4.94
Myohemoglobin	50.26 ng/mL	<72
D-dimer	2.2 mg/L FEU	<1.15
Prothrombin time	15.2 s	9.6–12.8
Thrombin time	19.4 s	14–22
International normalized ratio	1.29	0.88–1.15
Fibrinogen	1.91 g/L	2–4
Fibrin degradation products	5.7 mg/L	<5

**Figure 1:**
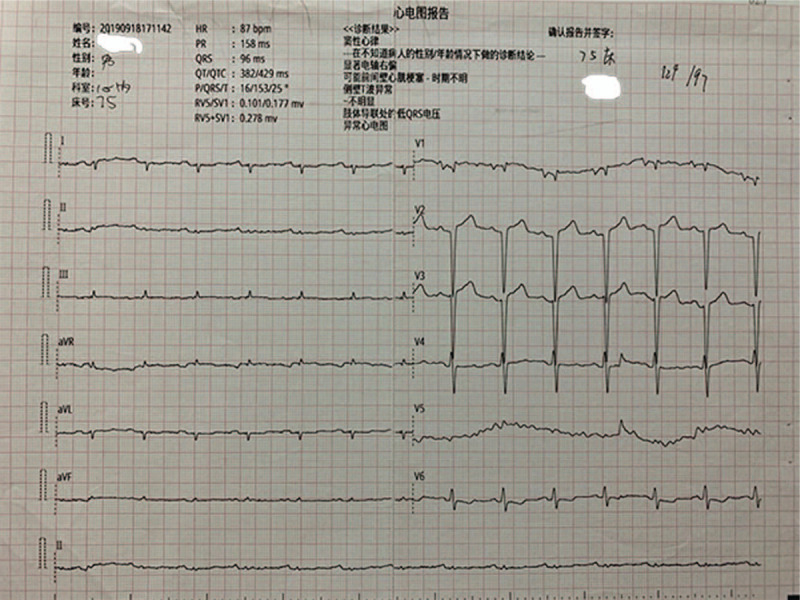
Electrocardiogram. Electrocardiogram revealed low voltages in limb leads, QT internal prolongation, Q-wave on V1 and V2 leads both on admission and in hospital.

**Figure 2:**
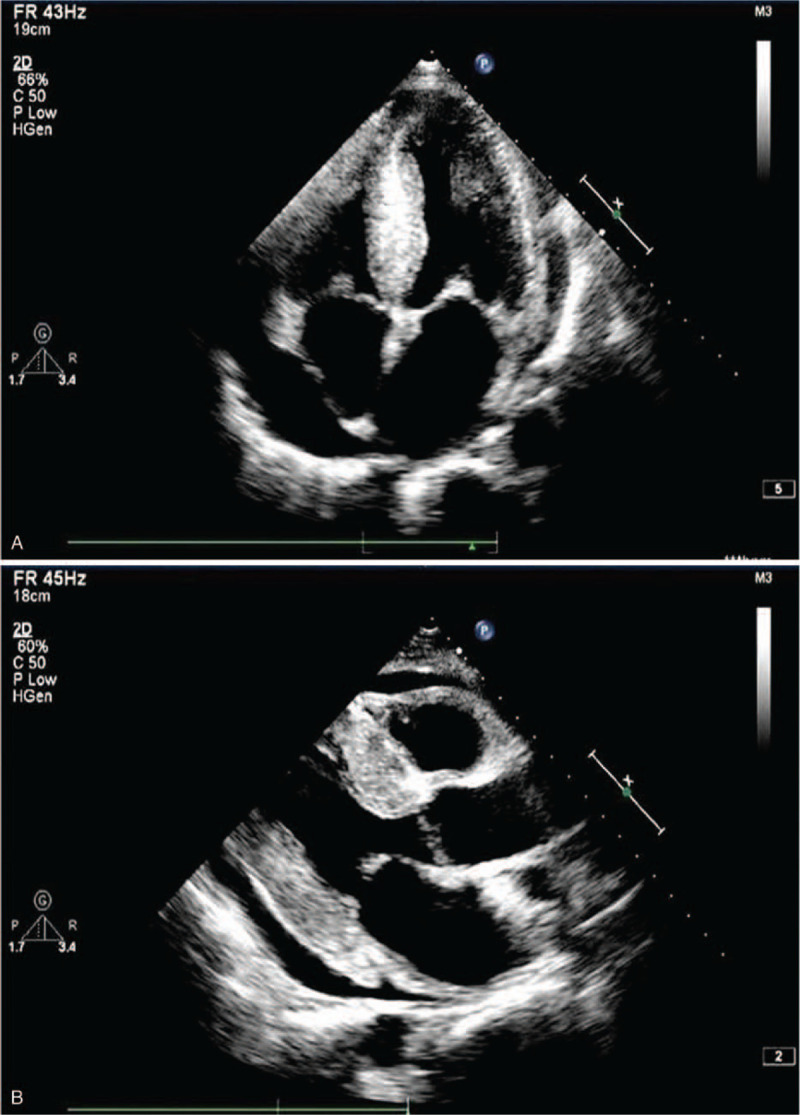
A. Apical Echocardiology. Apical 4 chamber view on echocardiology showed left ventricle hypertrophy, left atrium enlargement, and pericardial effusion. B. Parasternal long axis view echocardiology. Parasternal long axis view echocardiology showed left ventricle concentric hypertrophy and pericardial effusion.

**Figure 3:**
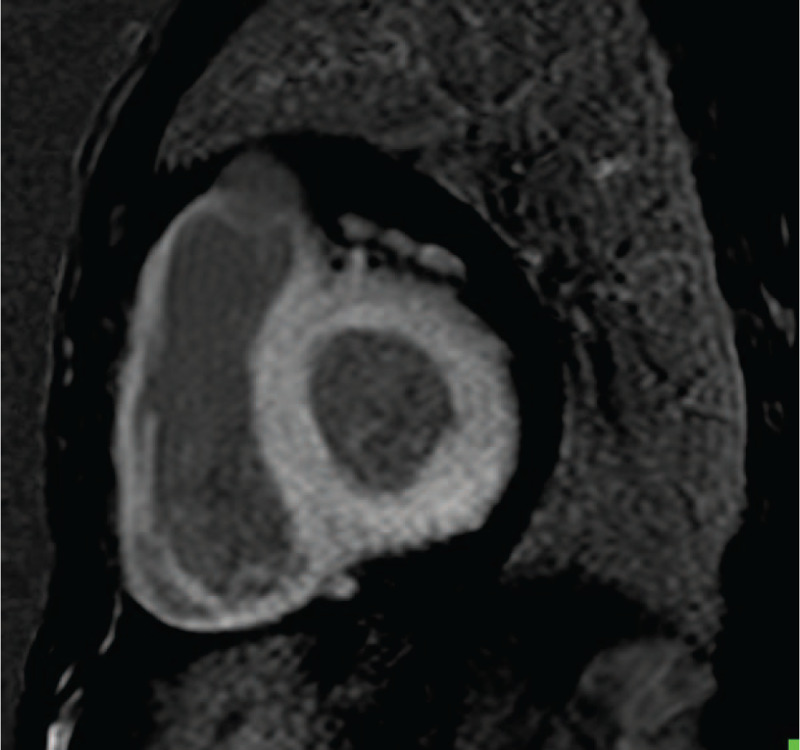
CMR. Cardiac magnetic resonance imaging (CMR) showed a diffuse transmural late gadolinium enhancement (LGE) in ventricular walls. Cardiac magnetic resonance imaging showed a diffused transmural LGE in both ventricular walls which suggested cardiac amyloidosis.

**Figure 4:**
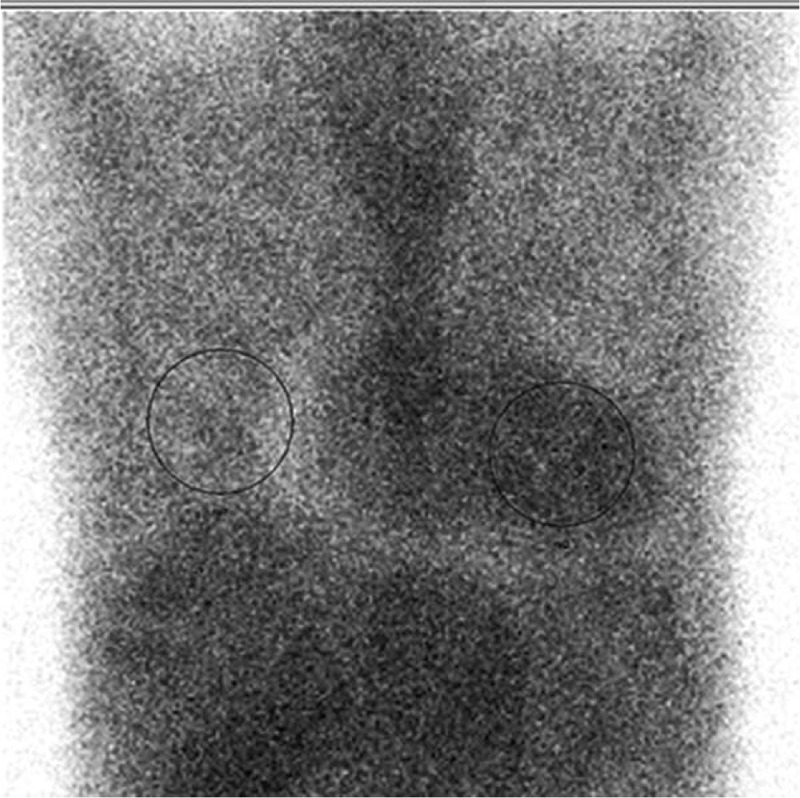
Tc-99m PYP scintigraphy. Intensive Tc-99m PYP uptake on nuclear scintigraphy. Tc-99m PYP scintigraphy revealed an intensive cardiac tracer uptake. The heart radio activity is equal to that of the ribs (grade 2).

Electrocardiogram revealed low voltages in limb leads, QT internal prolongation, Q-wave on V1 and V2 leads. Figure 1. Echocardiography demonstrated an increased thickness of left ventricular wall (18 mm), decreased left ventricular systolic function with LVEF of 27.5% (>50%), an enlarged right ventricle, enlarged atrium, and moderate pericardial effusion. Figure 2A and B. Cardiac magnetic resonance imaging (CMR) showed a diffused transmural late gadolinium enhancement (LGE) in both ventricular walls which suggested cardiac amyloidosis. Figure 3. Tc-99m PYP scintigraphy revealed an intensive cardiac tracer uptake (grade 2, heart equal to the ribs) and the heart-to-contralateral (H/CL) ratio was 1.65. Figure 4.

The patient underwent sub endocardial myocardial biopsy through right heart catheterization, and multiple samples were obtained from right atrium septum. The pathologic study on microscopy revealed a moderate intercellular fibrosis without inflammation, with ambiguity Congo red strain. Immunofluorescence demonstrated a positive lambda light chain and negative IgG, IgA, IgM, and kappa light chain. Immunohistochemistry revealed a positive lambda light chain, and negative kappa light chain and TTR, as well as negative CD3, CD8, CD68 etc. Figure 5 A–C. The pathological diagnosis was cardiac amyloidosis light chain (lambda).

**Figure 5:**
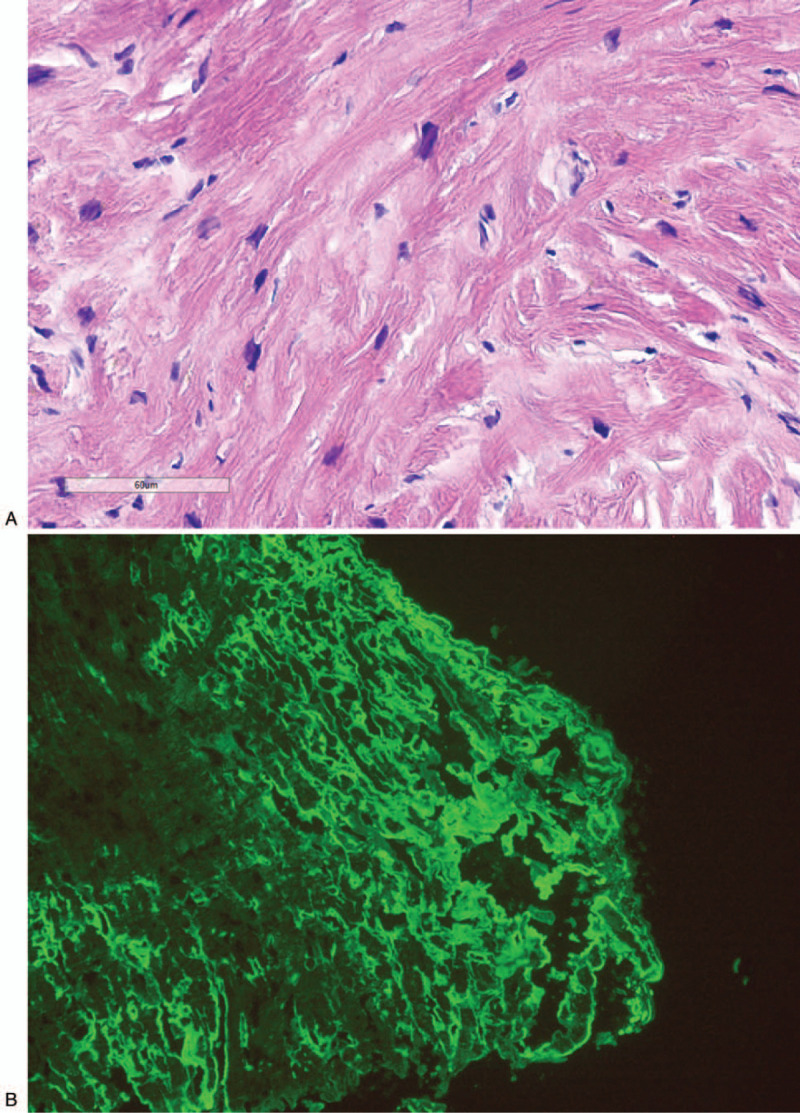
A Pathology-Congo red strain in myocardium (HE 400×). Ambiguity Congo red strain in myocardium on microscopy (HE 400×). B. Immunofluorescence in myocardium (lambda 200×). Positive lambda light chain in myocardium on immunofluorescence (lambda 200×). C. Immunohistochemistry in myocardium (lambda 200×). Positive lambda light chain in myocardium on immunohistochemistry (lambda 200×).

**Figure 5 (Continued):**
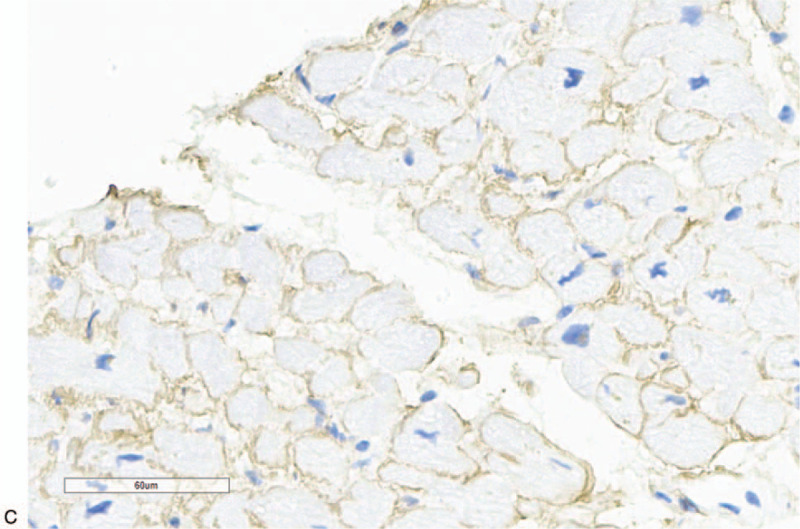
A Pathology-Congo red strain in myocardium (HE 400×). Ambiguity Congo red strain in myocardium on microscopy (HE 400×). B. Immunofluorescence in myocardium (lambda 200×). Positive lambda light chain in myocardium on immunofluorescence (lambda 200×). C. Immunohistochemistry in myocardium (lambda 200×). Positive lambda light chain in myocardium on immunohistochemistry (lambda 200×).

The patient was diagnosed as cardiac amyloidosis light chain (lambda) with severe heart failure and stroke. He was administered with furosemide 40 mg qd, torasemide 20 mg qd, spirolactone 20 mg qd, potassium chloride 10 mL per 500 mL urine, atorvastatin calcium tablet 20 mg qd, aspirin enteric-coated tablets 100 mg qd during the 2-weeks in-hospital. He died of heart failure around 2 months later after discharge.

## 3 Discussion

The patient complained of effort intolerance, tachycardia, short of breath, leg swelling over 1 year. Echocardiology demonstrated a decreased LV systolic function. Cardiac marker revealed an elevated BNP and Troponin-T. He was diagnosed with heart failure and estimated New York Heart Association Class III. Echocardiology demonstrated thickness of the left ventricle wall with a decreased left ventricular diastolic function. CMR showed a diffuse transmural LGE in both ventricular walls. Both echocardiology and CMR suggested a possible cardiac amyloidosis and need for further evaluation.^[4]^

Serum FLCs and Tc-99m PYP scintigraphy are 2 key modalities for differentiating AL from ATTR. Serum FLCs testing revealed a normal serum free kappa light chain and an elevated serum free lambda light china, and a decreased free kappa/lambda ratio. Serum and urine immunofixation electrophoresis demonstrated a positive lambda light chain-M protein. Both FLCs and immunofixation indicate the cardiac amyloidosis light chain.^[4]^ However, the patient presented a positive Tc-99m PYP scanning which is a feature of ATTR.

The patient underwent a subendocardial myocardial biopsy, and the pathological study on microscopy revealed cardiac amyloidosis. Immunohistochemistry revealed a positive lambda light chain, and negative kappa light chain and TTR, which indicated a light chain cardiac amyloidosis.

Based on the FLCs, pathology of myocardial biopsy, and Tc-99m PYP studies, the patient was diagnosed AL; and we considered a false positive Tc-99m PYP scanning. False positive Tc-99m PYP/DPD/HMDP scintigraphy were reported in patient with light chain cardiac amyloidosis.^[5,6]^ A recent expert consensus recommendations indicated that to diagnose ATTR amyloidosis with positive Tc-99m DPD/PYP/HMDP scan requires the absence of a clonal plasma cell process by free light chains with serum and urine immunofixation as an essential component.^[4]^ Similar to ATTR cardiac amyloidosis, the principle of false positive Tc-99m PYP/DPD/HMDP scintigraphy in patient with light chain cardiac amyloidosis may be calcification. Stats and Stone^[8]^ demonstrated a greater density of small microcalcifications in 8 cases of ATTR amyloidosis (mean = 16.8 per 200× field) than in 7 cases of AL amyloidosis(6.5 per 200× field) with von Kossa calcium stains in the samples of endomyocardial biopsies. It means that the AL amyloidosis also has small micro calcifications which may be a reason for false positive Tc-99m DPD/PYP/HMDP scintigraphy.

Technique aspects of the false positive Tc-99m PYP/DPD/HMDP scintigraphy include the following. The first aspect is that the 1-hour Tc-99m PYP scan may have more false positives rate than the 3-hour delay Tc-99m PYP scintigraphy. The second aspect is the blood pool may interference the score/grade. The third aspect is the possible increase of the heart score because of metabolism disorders, like osteoporosis. So, we need to do 3-hour delay or SPECT in patients with a grade 2 positive Tc-99m PYP scintigraphy, and correctly score the heart image.

Also, the recommendations for possible cardiac amyloidosis on PET using 18F-florbetapir or 18F- PET are target to background (LV myocardium to blood pool) ratio >1.5, or retention index >0.030/min.^[9,10]^ We did not do 18F-florbetapir or 18F-florbetaben studies. Electrophoresis showed a normal M-protein of 6.60% (10.6–23.5%), which ruled out multiple myeloma, smoldering multiple myeloma, and monoclonal gammopathy of undetermined significance (MGUS). The limitation of the study is that we did not identify the pre-protein with the myocardial biopsy samples with mass spectrometry; and we did not do 3-hour delay scan or SPECT to rule out the interference by the blood pool.

## 4 Conclusions

False positive Tc-99m PYP scintigraphy may rarely presented in patients with cardiac amyloidosis light chain. So, the clonal plasma cell process based on the FLCs and immunofixation is a base to rule out AL cardiac amyloidosis when we interpret a positive Tc-99m PYP scintigraphy.

## Author contributions

**Conceptualization:** Gongshun Tang.

**Data curation:** Gongshun Tang, Yu Zeng.

**Formal analysis:** Poterucha J. Timothy, Qing Zhang, Yucheng Chen, Yujia Liang.

**Funding acquisition:** Gongshun Tang.

**Investigation:** Gongshun Tang, Qing Zhang, Yucheng Chen, Hangyu Xie, Ke Wan, Yujia Liang, Juncheng Chen.

**Resources:** Gongshun Tang, Yucheng Chen, Ke Wan, Yujia Liang.

**Supervision:** Gongshun Tang.

**Writing – original draft:** Yu Zeng, Poterucha J. Timothy, Hangyu Xie.

**Writing – review & editing:** Gongshun Tang, Einstein J. Andrew.
